# In this month’s Bulletin

**DOI:** 10.2471/BLT.18.000818

**Published:** 2018-08-01

In the editorial section, Akihiro Nishi et al. (514) discuss what’s needed to reduce Thailand’s annual road traffic death rate to less than 10 deaths per 100 000 people by 2020. Marc Bulterys and Saeed Sadiq Hamid (515) introduce new treatment guidelines for people with chronic hepatitis C virus infection. 

Sima Barmania (518–519) investigates efforts to improve the health impacts of airports on passengers, staff and people living nearby.

Danius Puras talks to Fiona Fleck (520–521) about persuading governments to protect the rights of children with mental disabilities. 

## Australia

### Tracking annual variations

Kaitlyn Vette et al. (558–567) set thresholds for assessing the severity of pandemic influenza.

## Central African Republic

### Making up for lost years

Nicolas Peyraud et al. (540–547) describe a post-conflict vaccination campaign.

## China

### Causes of stillbirth

Tao Xiong et al. (531–539) study the impact of untreated hypertension in pregnancy. 

### Second pregnancy outcomes

Yi Mu et al. (548–557) track prior caesarean sections and likelihood of vaginal birth.

### Identifying women at high risk of breast cancer

Li Sun et al. (568–577) assess cost–effectiveness of a new screening programme. 

## Democratic Republic of the Congo

### Improving tuberculosis case-finding

Emmanuel André et al. (522–530) enlist patients to find other people with untreated infections.

## Samoa

### Detecting and treating noncommunicable diseases 

Caroline Bollars et al. (578–583) describe an adaptation of WHO’s recommended interventions.

## Global

### Reducing civilian hazards 

Jawad and Youssef Fares (584–585) call for better compliance with a cluster munitions treaty.

### Beyond a national response

Arian Hatefi et al. (586–588) consider noncommunicable diseases in terms of global susceptibility.

